# Network-Based Association Study of Obesity and Type 2 Diabetes with Gene Expression Profiles

**DOI:** 10.1155/2015/619730

**Published:** 2015-07-27

**Authors:** Siyi Zhang, Bo Wang, Jingsong Shi, Jing Li

**Affiliations:** ^1^Department of Bioinformatics & Biostatistics, School of Life Science and Biotechnology, Shanghai Jiao Tong University, Shanghai 200240, China; ^2^National Clinical Research Center of Kidney Disease, Jinling Hospital, Nanjing University School of Medicine, Nanjing 210016, China; ^3^Shanghai Center for Bioinformation Technology, Shanghai 201203, China

## Abstract

The increased prevalence of obesity and type 2 diabetes (T2D) has become an important factor affecting the health of the human. Obesity is commonly considered as a major risk factor for the development of T2D. However, the molecular mechanisms of the disease relations are not well discovered yet. In this study, the combination of multiple differential expression profiles and a comprehensive biological network of obesity and T2D allowed us to identify and compare the disease-responsive active modules and subclusters. The results demonstrated that the connection between obesity and T2D mainly relied on several pathways involved in the digestive metabolism, immunization, and signal transduction, such as adipocytokine, chemokine signaling pathway, T cell receptor signaling pathway, and MAPK signaling pathways. The relationships of almost all of these pathways with obesity and T2D have been verified by the previous reports individually. We also found that the different parts in the same pathway are activated in obesity and T2D. The association of cancer, obesity, and T2D was identified too here. As a conclusion, our network-based method not only gives better support for the close connection between obesity and T2D, but also provides a systemic view in understanding the molecular functions underneath the links. It should be helpful in the development of new therapies for obesity, T2D, and the associated diseases.

## 1. Introduction

A sedentary life-style coupled with calorie-dense dietary behavior of contemporary human causes the accumulation of body fat. In the past decades, the prevalence of obesity increased rapidly in industrialized societies with its undesirable consequences such as type 2 diabetes (T2D), high blood pressure, and heart diseases [1]. The latest National Health and Nutrition Examination Survey (NHANES) program estimated that the prevalence of obesity (defined as a body mass index greater than 30) in adults has reached 36% in the United State [2], while the global incidence of diabetes mellitus is expected to increase to 366 million cases by the year 2030 [3].

Obesity is commonly considered as a major risk factor for the development of T2D. It has been reported that the altered glucose and lipid metabolism in liver, skeletal muscles, and adipose tissues with the disorganized insulin signals lead to the systemic and chronic inflammation [4, 5]. They also recognized that the obesity-caused metabolic inflammation could connect obesity to the insulin resistance (IR), which is associated with T2D [6, 7]. And very few disease genes have been reported in both obesity and diabetes, such as PPARG [8, 9] and UCP3 [10]. However, the molecular mechanism in the association between obesity and diabetes is still far from being fully understood.

Recently biological network and high-throughput gene expression data are emerging as useful resources in revealing the molecular mechanisms of complex disease [11–14]. In this study, using genome-scale gene differential expression profiles and an integrated biological network of obesity and T2D, which contained the information in protein-protein interactions, transcriptional regulation, and metabolic pathways, we identified and compared the gene network and the active subnetworks in pathology between obesity and T2D in order to provide novel insight to understand the molecular association between them.

## 2. Materials and Methods

### 2.1. Disease Genes and Gene Links

26 obesity genes and 34 T2D genes were collected from the Online Mendelian Inheritance in Man (OMIM) [15] as the seed genes. Three seed genes were common between obesity and T2D. The experimentally validated protein-protein interactions and transcriptional regulation of these seed genes and their neighbors were extracted from the human protein interaction database HPRD [16] (Release 9) and TRANSFAC database [17] (Release 2013.2), as well as from 29 KEGG pathways [18] (Release 71.1, September 1, 2014) enriched by the known obesity and T2D genes. In the interaction file downloaded from KEGG, only the PPrel (protein-protein interaction) and GErel (gene expression interaction) were extracted and added to this study.

### 2.2. Gene Expression Profiles and Processing

We collected twelve microarray datasets totally in case-control design from the NCBI Gene Expression Omnibus (GEO) [19] for obesity (GSE10946, GSE15653, GSE29718, GSE48964, GSE9624) and T2D (GSE18732, GSE13760, GSE20966, GSE23343, GSE25724, GSE38396, and GSE38642). All of these datasets were curated and reported in the GEO Datasets (GDS). Each dataset was required to have at least three samples for both case and control groups. And the samples from these patients who suffered both obesity and diabetes were excluded.

The preprocessing of microarray data was conducted by the RMA [20–22] integrative method, and the statistical analysis of gene differential expression was computed by the linear models and empirical Bayes methods [23]. And then the *P* values of each gene were obtained.

### 2.3. Identification of Active Modules and Subclusters

From the gene network of obesity and T2D, we used the jActiveModules [24] and multiple gene expression profiles to find the active gene modules showing significant changes in expression in disease/normal conditions. The jActiveModules (Version 1.8) is a widely used method for identifying active modules integrating multiple gene differential expression datasets. In the algorithm of jActiveModules [24], the *P* values of each gene in a subnetwork in a single condition are transformed into one standard normal *z*-score by the binomial order statistic. The highest score obtained in multiple experiments is recorded as the final score for a subnetwork. Higher *z*-score represents more significant expression changes. Here the top 5 scoring modules of obesity and T2D were enumerated separately by jActiveModules with default parameters in Cytoscape [25].

**Figure 1:**
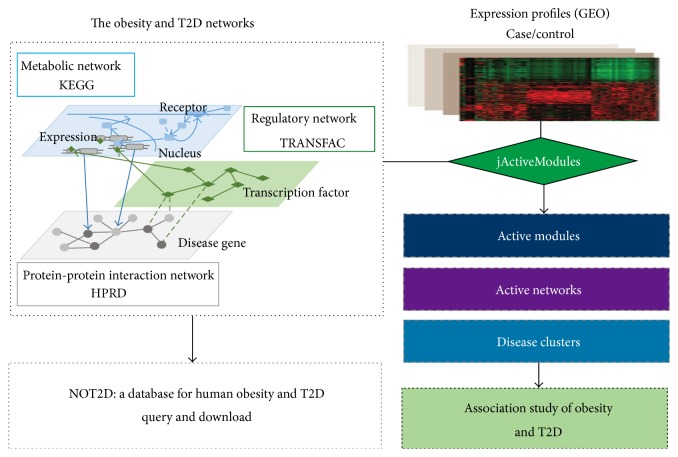
The identification of active modules and subclusters in human obesity and T2D. In the workflow, the disease genes of obesity and T2D were obtained from OMIM, and the interacting neighbors were collected from HPRD, KEGG, and TRANSFAC to construct a gene network of obesity and T2D (NOT2D). Multiple differential expression datasets in case/control design for obesity or T2D were integrated with the NOT2D network using jActiveModules method in order to identify the active modules and clusters. And finally a network association study of obesity and T2D was performed based on these active modules and clusters.

In order to further identify the subclusters with tight topology structures, we decomposed the active modules and the disease seed genes into several subclusters. As a result, ten and seven subclusters were identified by the MCODE method [26] for obesity and T2D, respectively. The workflow was illustrated in Figure 1.

## 3. Results and Discussion

### 3.1. The NOT2D Network

**Figure 2:**
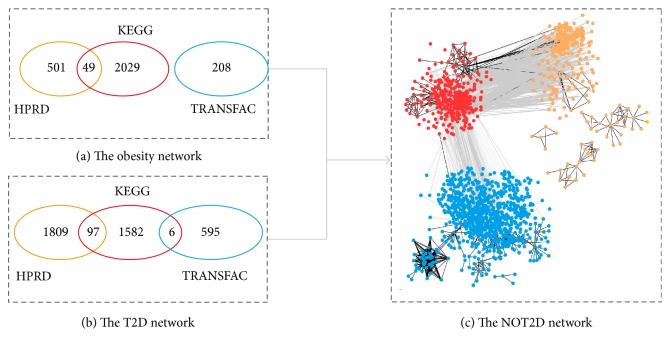
The edges and nodes distributions in the NOT2D network. (a) and (b) show the numbers of edges derived from the HPRD, KEGG, and TRANSFAC in the obesity and T2D networks. (c) displays the shared genes (red) and the specific genes of the obesity (orange) and T2D (blue) network.

Through collecting the protein interactions and transcriptional regulation data of the known genes of human obesity and T2D and their interacting neighbors from HPRD, TRANSFAC, and KEGG pathways, we compiled a multi-level biological network of human obesity and T2D called NOT2D (gene network of obesity and type 2 diabetes) (Figure 1). As shown in Figure 2, the majority of links in the obesity network were obtained from KEGG database while HPRD and KEGG databases contributed almost equally to the T2D network. Very few interactions are reported by two or more data sources. Finally, there are 606 nodes and 2907 edges in the obesity network and 1211 nodes and 4089 edges in the network of T2D. Among 7170 unique edges in the NOT2D network, there are 6229 protein-protein interactions and 941 gene regulatory links. 374 out of 1443 nodes in the NOT2D network are shared by obesity and T2D. Surprisingly the average degree of the shared genes is 19.2, which is more than twice the average value of 9.5 in the whole NOT2D network.

The interaction data of the NOT2D network can be downloaded at http://lilab.life.sjtu.edu.cn:8080/NOT2D/.

### 3.2. Topology and Function of the Active Subclusters

By combing the differential expression profiles from multiple datasets and the NOT2D network, the top 5 scoring active modules were identified in the obesity samples as well as in T2D. And then the top 5 active modules and the seed genes were merged into an active network for both obesity and T2D.

**Figure 3:**
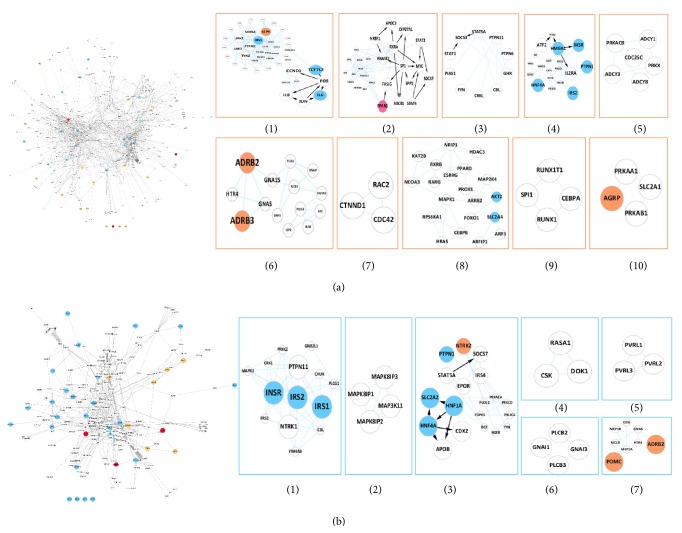
The obesity or T2D active networks and subclusters. (a) The active networks and ten subclusters of obesity. (b) The active networks and seven subclusters of T2D. The known seed genes of obesity (orange) and T2D (blue) are highlighted, as well as the shared genes (pink). The important genes in subclusters are also shown by bigger node sizes. The black arrows indicate the regulatory interactions while the blue links are protein interactions.

To better understand the biological processes or molecular function underneath the active gene network of obesity or T2D, we decomposed the active networks into 10 obesity clusters and 7 T2D functional clusters by the MCODE method, of which from 3 to 43 genes were contained. The topology structures of these clusters were displayed in Figure 3.

As an example, a seed gene IL6 in the obesity cluster 1 is regulated by JUN and FOS, which is secreted by M1 macrophages, and often takes effect in promoting obesity-associated inflammation which aggravates the progression of metabolic complications, such as cardiovascular disease and insulin resistance [27]. Specially, as a member of lipid-sensing peroxisome proliferator-activated receptor (PPAR) family, PPAR-*γ* in obesity cluster 2 is a common disease gene for both obesity and T2D, which is a master regulator in adipocyte differentiation and whole-body insulin sensitivity [28, 29], while in T2D cluster 1, the insulin receptor (INSR) and insulin receptor substrate-1/2 (IRS1/2) were identified many years ago as key factors for insulin pathways to keep the carbohydrate homeostasis [30–32]. In addition, the proopiomelanocortin (POMC) and the agouti related protein homolog (AGRP) in T2D cluster 7 play a vital role in the balance of food intake and energy expenditure, through the generated neuronal and hormonal signals [33–35].

It can be found from Figure 3 that some active clusters did not contain any seed genes of obesity or/and T2D, such as obesity clusters 3, 5, 7, and 9 and T2D clusters 2, 4, 5, and 6. We inferred that most of these active clusters would be involved in the important processes related to obesity or T2D. In order to verify our point, the KEGG enrichment analysis was performed to all of the active subnetworks by the WebGestalt [36] (BH adjusted *P* value < 0.05).

**Figure 4:**
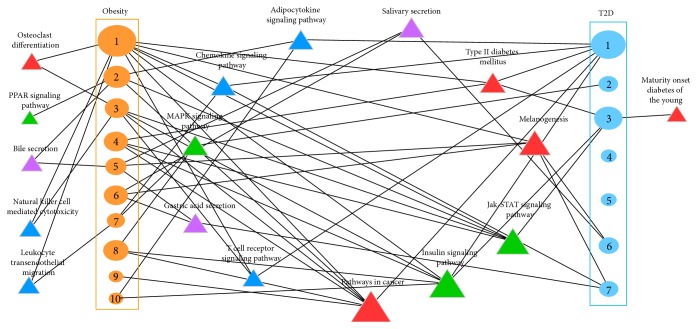
The KEGG pathways enriched in obesity and T2D clusters. The enriched pathways of the obesity (orange ellipse) and T2D (blue ellipse) clusters are classified into three regulatory groups (metabolic, immune response, and signaling) and one disease-related group, which were highlighted by the colored triangles (purple, blue, red, and grass green). The size of ellipse represents the number of genes in the subclusters and the triangle size is proportional to the number of the links with obesity and T2D subclusters.

As a result, there are 16 and 12 pathways significantly enriched in these obesity and T2D active clusters with or without the seed genes (Figure 4). It verified our conjecture very well as almost all these enriched pathways have been reported for their connection with the development of obesity and/or T2D, such as PPAR signaling pathway [9], insulin signaling pathway [37], and MAPK signaling pathway [38]. For instance, the PPAR signaling pathway is enriched in active cluster 2 of obesity, which has a vital function in adipocyte proliferation and differentiation in liver, muscle and adipose tissues [9]. The PPARs not only regulate lipid, carbohydrate, and amino acid metabolism, but also play an important role in systemic insulin sensitization through the combined effects of the production of adiponectin and reduction of lipotoxicity [9].

The insulin signaling pathway was enriched in obesity cluster 3 containing no seed gene [37]. Three genes (CRKL, CBL, and SOCS3) in this cluster paly roles in the Insulin signaling pathway, and SOCS3 gene has been reported for its inhibition of insulin signals of the adipose tissues [37]. The insulin signals have marked function of blood sugar reduction and improving sugar tolerance, which result in the development of obesity and T2D [38]. The obesity-associated insulin resistance is a major risk factor for type 2 diabetes and cardiovascular disease [39]. As a second example, all of the genes in T2D cluster 2 are the important members of MAPKs family which participates in the enriched MAPK signaling pathway. MAPK signaling pathway, which can be activated by insulin, is required for an array of metabolic events. The excessive activation of MAPKs is associated with detrimental effects on obesity and diabetes that contribute to disease progression [40]. These genes detected in the active clusters might be new candidate disease genes or biomarkers for obesity and T2D.

### 3.3. The Network Association between Obesity and T2D

There are lots of evidences demonstrating the strong connection between obesity and T2D. But so far only three genes (ENPP1, PPARG, and UCP3) are common in 26 obesity genes and 34 T2D genes annotated in the OMIM database. In order to explore the molecular association of obesity and T2D at the level of biological network, we constructed and compared the disease networks of obesity and T2D instead of the individual genes. As shown in Figure 2(b), the percentage of the shared nodes in the obesity and T2D networks is increased dramatically to 26% while only 5% genes are shared at individual gene level. Additionally, we found in Figure 2(c) that the hub genes play critical roles in linking obesity and T2D since the average degree of the shared genes is significantly higher than the remains in the NOT2D network.

**Figure 5:**
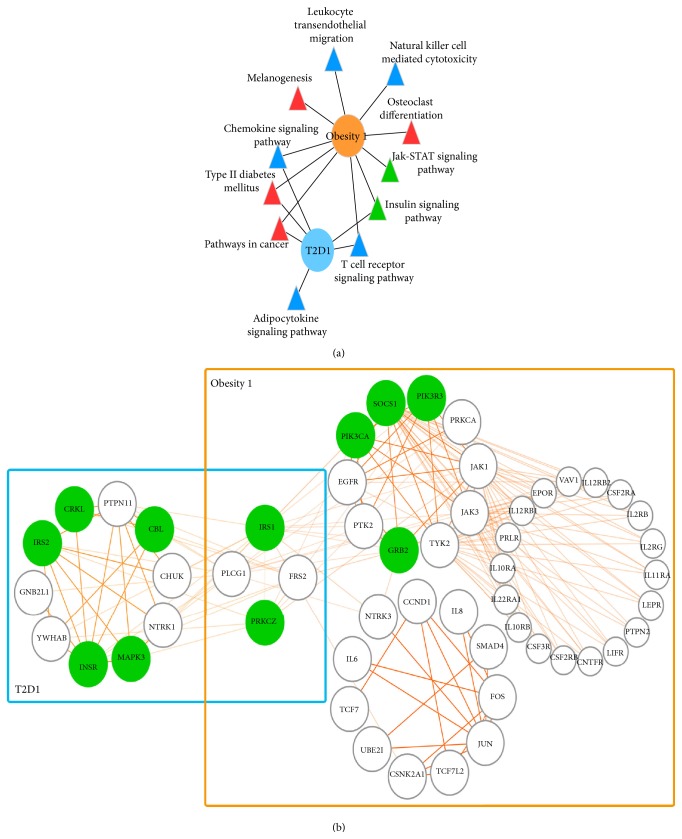
Functional connections brought by the active clusters between T2D and obesity. (a) The connecting pathways in the obesity (orange ellipse) and T2D (blue ellipse) cluster 1. The enriched pathways of the immune response, signaling, and disease-related groups were highlighted by the colored triangles (blue, red, and grass green). (b) The graph of T2D cluster 1 and obesity cluster 1. The genes involved in insulin signaling pathway were highlighted in green.

Whereafter, by applying the differential gene expression data in case/control design into the network, we identified the active gene networks and the subclusters of obesity and T2D. In the results, the node overlap of the active subclusters in obesity and T2D is very rare. However, the following functional analysis revealed that most of the pathways activated in obesity and T2D are the same (Figure 4). Eleven of the twelve activated pathways identified in obesity were also reported in T2D. Given an example, pathways in cancer, insulin signaling pathway, and other three pathways are enriched not only in obesity cluster 1 but also in T2D cluster 1, even though the gene overlap of these two clusters is very few (Figure 5). When we looked at more in insulin signaling pathway that regulating the whole glucose and lipid metabolism, and found that the activated parts of this pathway in obesity and T2D are different distinctly. Seven genes in the T2D cluster 1 and six genes in obesity cluster 1 are involved in insulin signaling pathway, but only two genes are the same. This result suggests that the association between obesity and T2D depends on the coactivated gene clusters or pathways rather than a few individual disease genes.

In general, the activated pathways connecting obesity and T2D mainly fall into four categories: digestive metabolism system, immune system, signaling transduction, and disease related pathways. Two digestive metabolism pathways, gastric acid secretion [41] and salivary secretion [42], are essential for digestion and absorption of protein, fats, and fat-soluble vitamins in the small intestine. In addition, the dysregulation of the energy metabolism may induce the accumulation of fats that lead to the obesity finally [43].

The responses of immune system also held a very important part in linking obesity and T2D, such as chemokine and T cell receptor signaling pathways. Previous studies reviewed that the nutrient and energy overload can induce the accumulation of adipose tissues and triggered the inflammatory cytokine expression; also the chronicity metabolic inflammation of fat cells could certainly change the energy intake and expenditure and insulin sensitivity states [7, 44]. Some chemokines are considered proinflammatory and can be induced during an immune response to recruit cells of the immune system to a site of infection [45]. The proinflammatory cytokine TNF alpha has been implicated as a link between obesity and insulin resistance [46].

Adipocytokines have been recently defined as soluble mediators derived mainly from adipocytes, in the interaction between adipose tissue, inflammation, and immunity. Thereby adipose tissue has been redefined as a key component not only of the endocrine system, but also of the immune system [47].

Some signal transduction pathways are also involved in the connection between obesity and T2D. The Jak-STAT signaling pathway is the principal signaling mechanism for a wide array of cytokines and growth factors, especially the critical role in regulating leukocyte maturation and activity [48], while MAPK signaling pathway is involved in cell proliferation, differentiation, and migration [49]. Obesity pathogenesis can be caused by mutations in the MC4R gene in MAPK signaling pathway [50].

Epidemiologic studies have indicated that diabetes and obesity are linked to an increased risk of certain cancers in association with higher levels of insulin and insulin-like growth factor 1 [51]. Newer therapies targeting the insulin and IGF1 systems are being developed for use in cancer therapy [52].

Based on our study and the previous reports, it is suggested that the association of obesity and T2D can be described as a “step-by-step” process in some way. At the initial stage, the abnormal accumulation of food intake and adipose tissues triggers the inflammatory cytokine expression and persistent immune reactions of fat cells. And then obesity-induced inflammation responses change the insulin sensitivity state and lead to the occurrence of insulin resistance consequently, which marks the transformation from obesity to obesity-related T2D.

But how these pathways interplay underlies the pathophysiology of obesity and T2D is still a big challenge. And the risk of false negative in the biological network also exists. Even with these challenges, network-based systems biology is increasingly attracting much attention from communities of both experimental and computational biologists and is expected to revolutionize our understanding of complicated disease as a whole. The methodologies and techniques of systems biology have been applied to analyzing the molecular mechanisms of complex diseases and provided new solutions for preventing and curing the diseases [53]. With vast amounts of omics data generated, the method still provides a new perspective for the future disease association studies.

### 3.4. NOT2D, a Database for Human Obesity and T2D

**Figure 6:**
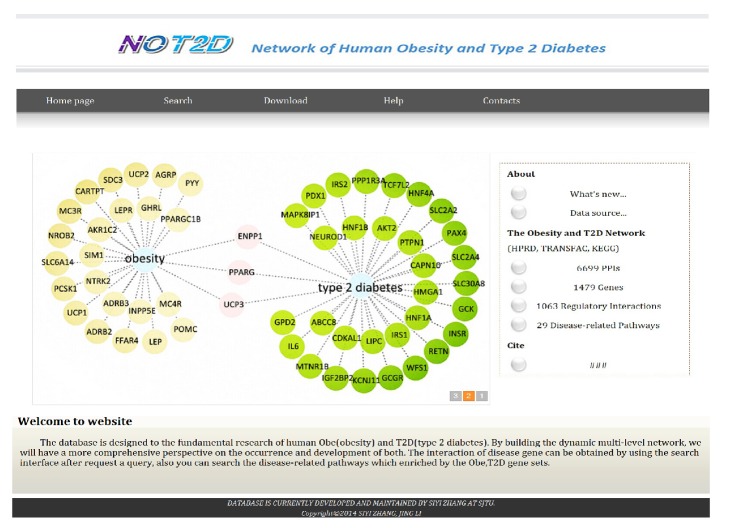
The snapshot of the NOT2D database.

We constructed the NOT2D (network of human obesity and T2D) database to store the known disease genes and the interaction or regulatory links that are related to obesity and T2D (Figure 6). The obesity or T2D related genes, pathways, and networks can be accessed and downloaded from the website http://lilab.life.sjtu.edu.cn:8080/NOT2D/.

## 4. Conclusion

We studied the association between obesity and T2D by combing the gene expression profiles and the comprehensive biological network including both protein-protein interactions, metabolic and regulatory links. This study revealed that the connection of obesity and T2D mainly relied on several pathways involved in the digestive metabolism, immunization, and signal transduction. Our network-based association analysis provided better support and systematic explanation for the close connection between obesity and T2D than in view of individual gene.

## References

[1] Guilherme A., Virbasius J. V., Puri V., Czech M. P. (2008). Adipocyte dysfunctions linking obesity to insulin resistance and type 2 diabetes. *Nature Reviews Molecular Cell Biology*.

[2] Flegal K. M., Carroll D., Kit B. K., Ogden C. L. (2012). Prevalence of obesity and trends in the distribution of body mass index among US adults, 1999–2010. *The Journal of the American Medical Association*.

[3] Patel D. K., Kumar R., Laloo D., Hemalatha S. (2012). Diabetes mellitus: an overview on its pharmacological aspects and reported medicinal plants having antidiabetic activity. *Asian Pacific Journal of Tropical Biomedicine*.

[4] Ahmad J., Ahmed F., Siddiqui M. A., Hameed B., Ahmad I. (2006). Inflammation, insulin resistance and carotid IMT in first degree relatives of north Indian type 2 diabetic subjects. *Diabetes Research and Clinical Practice*.

[5] Pickup J. C., Crook M. A. (1998). Is Type II diabetes mellitus a disease of the innate immune system?. *Diabetologia*.

[6] Reaven G. M. (1998). Insulin resistance and human disease: a short history. *Journal of Basic and Clinical Physiology and Pharmacology*.

[7] Gregor M. F., Hotamisligil G. S. (2011). Inflammatory mechanisms in obesity. *Annual Review of Immunology*.

[8] Kilpeläinen T. O., Lakka T. A., Laaksonen D. E. (2008). SNPs in PPARG associate with type 2 diabetes and interact with physical activity. *Medicine and Science in Sports and Exercise*.

[9] Evans R. M., Barish G. D., Wang Y.-X. (2004). PPARs and the complex journey to obesity. *Nature Medicine*.

[10] Jia J.-J., Zhang X., Ge C.-R., Jois M. (2009). The polymorphisms of UCP2 and UCP3 genes associated with fat metabolism, obesity and diabetes: etiology and pathophysiology. *Obesity Reviews*.

[11] Goh K.-I., Cusick M. E., Valle D., Childs B., Vidal M., Barabási A.-L. (2007). The human disease network. *Proceedings of the National Academy of Sciences of the United States of America*.

[12] Stelzl U., Worm U., Lalowski M. (2005). A human protein-protein interaction network: a resource for annotating the proteome. *Cell*.

[13] Harbison C. T., Gordon D. B., Lee T. I. (2004). Transcriptional regulatory code of a eukaryotic genome. *Nature*.

[14] Ravasi T., Suzuki H., Cannistraci C. V. (2010). An atlas of combinatorial transcriptional regulation in mouse and man. *Cell*.

[15] Hamosh A., Scott A. F., Amberger J., Bocchini C., Valle D., McKusick V. A. (2002). Online Mendelian Inheritance in Man (OMIM), a knowledgebase of human genes and genetic disorders. *Nucleic Acids Research*.

[16] Prasad T. S. K., Goel R., Kandasamy K. (2009). Human protein reference database—2009 update. *Nucleic Acids Research*.

[17] Matys V., Fricke E., Geffers R. (2003). TRANSFAC: transcriptional regulation, from patterns to profiles. *Nucleic Acids Research*.

[18] Kanehisa M., Goto S., Kawashima S., Okuno Y., Hattori M. (2004). The KEGG resource for deciphering the genome. *Nucleic Acids Research*.

[19] Barrett T., Troup D. B., Wilhite S. E. (2011). NCBI GEO: archive for functional genomics data sets-10 years on. *Nucleic Acids Research*.

[20] Irizarry R. A., Bolstad B. M., Collin F., Cope L. M., Hobbs B., Speed T. P. (2003). Summaries of affymetrix GeneChip probe level data. *Nucleic acids research*.

[21] Bolstad B. M., Irizarry R. A., Astrand M., Speed T. P. (2003). A comparison of normalization methods for high density oligonucleotide array data based on variance and bias. *Bioinformatics*.

[22] Irizarry R. A., Hobbs B., Collin F. (2003). Exploration, normalization, and summaries of high density oligonucleotide array probe level data. *Biostatistics*.

[23] Smyth G. K. (2004). Linear models and empirical Bayes methods for assessing differential expression in microarray experiments. *Statistical Applications in Genetics and Molecular Biology*.

[24] Ideker T., Ozier O., Schwikowski B., Siegel A. F. (2002). Discovering regulatory and signalling circuits in molecular interaction networks. *Bioinformatics*.

[25] Shannon P., Markiel A., Ozier O. (2003). Cytoscape: a software Environment for integrated models of biomolecular interaction networks. *Genome Research*.

[26] Bader G. D., Hogue C. W. V. (2003). An automated method for finding molecular complexes in large protein interaction networks. *BMC Bioinformatics*.

[27] Chawla A., Nguyen K. D., Goh Y. P. S. (2011). Macrophage-mediated inflammation in metabolic disease. *Nature Reviews Immunology*.

[28] Kliewer S. A., Forman B. M., Blumberg B. (1994). Differential expression and activation of a family of murine peroxisome proliferator-activated receptors. *Proceedings of the National Academy of Sciences of the United States of America*.

[29] Rosen E. D., Walkey C. J., Puigserver P., Spiegelman B. M. (2000). Transcriptional regulation of adipogenesis. *Genes & Development*.

[30] Lin X., Taguchi A., Park S. (2004). Dysregulation of insulin receptor substrate 2 in beta cells and brain causes obesity and diabetes. *Journal of Clinical Investigation*.

[31] Brunetti A., Manfioletti G., Chiefari E., Goldfine I. D., Foti D. (2001). Transcriptional regulation of human insulin receptor gene by the high-mobility group protein HMGI(Y). *The FASEB Journal*.

[32] Bruning J. C., Gautam D., Burks D. J. (2000). Role of brain insulin receptor in control of body weight and reproduction. *Science*.

[33] van Rossum C. T. M., Pijl H., Adan R. A. H., Hoebee B., Seidell J. C. (2006). Polymorphisms in the NPY and AGRP genes and body fatness in Dutch adults. *International Journal of Obesity*.

[34] Krude H., Biebermann H., Luck W., Horn R., Brabant G., Grüters A. (1998). Severe early-onset obesity, adrenal insufficiency and red hair pigmentation caused by POMC mutations in humans. *Nature Genetics*.

[35] Delplanque J., Barat-Houari M., Dina C. (2000). Linkage and association studies between the proopiomelanocortin (POMC) gene and obesity in Caucasian families. *Diabetologia*.

[36] Wang J., Duncan D., Shi Z., Zhang B. (2013). WEB-based GEne SeT AnaLysis Toolkit (WebGestalt): update 2013. *Nucleic Acids Research*.

[37] Fröjdö S., Vidal H., Pirola L. (2009). Alterations of insulin signaling in type 2 diabetes: a review of the current evidence from humans. *Biochimica et Biophysica Acta—Molecular Basis of Disease*.

[38] Bost F., Aouadi M., Caron L., Binétruy B. (2005). The role of MAPKs in adipocyte differentiation and obesity. *Biochimie*.

[39] Qatanani M., Lazar M. A. (2007). Mechanisms of obesity-associated insulin resistance: many choices on the menu. *Genes and Development*.

[40] Gehart H., Kumpf S., Ittner A., Ricci R. (2010). MAPK signalling in cellular metabolism: stress or wellness?. *EMBO Reports*.

[41] Yao X., Forte J. G. (2003). Cell Biology of Acid Secretion by the Parietal Cell. *Annual Review of Physiology*.

[42] Turner R. J., Sugiya H. (2002). Understanding salivary fluid and protein secretion. *Oral Diseases*.

[43] Goran M. I., Goran M. I. (2000). Energy metabolism and obesity. *Medical Clinics of North America*.

[44] Kanneganti T.-D., Dixit V. D. (2012). Immunological complications of obesity. *Nature Immunology*.

[45] Mogensen T. H. (2009). Pathogen recognition and inflammatory signaling in innate immune defenses. *Clinical Microbiology Reviews*.

[46] Li Y., Ding L., Hassan W., Abdelkader D., Shang J. (2013). Adipokines and hepatic insulin resistance. *Journal of Diabetes Research*.

[47] Tilg H., Moschen A. R. (2006). Adipocytokines: mediators linking adipose tissue, inflammation and immunity. *Nature Reviews Immunology*.

[48] Schindler C. W. (2002). Series introduction. JAK-STAT signaling in human disease. *Journal of Clinical Investigation*.

[49] Chen Z., Gibson T. B., Robinson F. (2001). MAP kinases. *Chemical Reviews*.

[50] He S., Tao Y.-X. (2014). Defect in MAPK signaling as a cause for monogenic obesity caused by inactivating mutations in the melanocortin-4 receptor gene. *International Journal of Biological Sciences*.

[51] Gallagher E. J., Fierz Y., Ferguson R. D., LeRoith D. (2010). The pathway from diabetes and obesity to cancer, on the route to targeted therapy. *Endocrine Practice*.

[52] Cohen D. H., LeRoith D. (2012). Obesity, type 2 diabetes, and cancer: the insulin and IGF connection. *Endocrine-Related Cancer*.

[53] Villoslada P., Steinman L., Baranzini S. E. (2009). Systems biology and its application to the understanding of neurological diseases. *Annals of Neurology*.

